# McKeown esophagectomy with concomitant median arcuate ligament release in a case of esophageal cancer with celiac artery stenosis

**DOI:** 10.1186/s40792-022-01359-z

**Published:** 2022-01-07

**Authors:** Keita Hanada, Shigeru Tsunoda, Satoshi Ogiso, Tatsuto Nishigori, Shigeo Hisamori, Kazutaka Obama

**Affiliations:** 1 Division of Gastrointestinal Surgery, Department of Surgery, Graduate School of Medicine, Kyoto University, 54 Shogoin- Kawahara-cho, Sakyo-ku, Kyoto, 606-8507 Japan; 2 Division of Hepato-Biliary-Pancreatic Surgery and Transplantation, Department of Surgery, Graduate School of Medicine, Kyoto University, 54 Shogoin- Kawahara-cho, Sakyo-ku, Kyoto, 606-8507 Japan

**Keywords:** Esophageal cancer, Esophagectomy, Celiac artery stenosis, Median arcuate ligament, Doppler ultrasonography

## Abstract

**Background:**

The celiac artery stenosis due to compression by median arcuate ligament (MAL) has been reported in many cases of pancreaticoduodenectomy, but not in cases of esophagectomy. Recently, the celiac artery stenosis due to MAL or arteriosclerosis has been reported to be associated with the gastric tube necrosis or anastomotic leakage following Ivor–Lewis esophagectomy. Herein, we present the first reported case of esophageal cancer with celiac artery stenosis due to compression by the MAL successfully treated by McKeown esophagectomy and gastric tube reconstruction following prophylactic MAL release.

**Case presentation:**

A 72-year-old female patient was referred to our department for esophagectomy. The patient had received two courses of neoadjuvant chemotherapy with 5-FU and cisplatin for T2N0M0 squamous cell carcinoma of the middle esophagus. Preoperative contrast-enhanced computed tomography (CECT) showed celiac artery stenosis due to compression by the MAL. The development of collateral arteries around the pancreatic head was observed without evidence of aneurysm formation. The patient reported no abdominal symptoms. After robot-assisted esophagectomy with mediastinal lymphadenectomy, gastric mobilization, supra-pancreatic lymphadenectomy, and preparation of the gastric tube were performed under laparotomy. Subsequently, the MAL was cut, and released to expose the celiac artery. Improved celiac artery blood flow was confirmed by decreased pulsatility index on intraoperative Doppler sonography. The operation was completed with the cervical esophagogastric anastomosis following cervical lymphadenectomy. Postoperative CECT on postoperative day 7 demonstrated increased celiac artery patency. The patient had an uncomplicated postoperative course thereafter.

**Conclusions:**

Prophylactic MAL release may be considered in patients with celiac artery stenosis due to compression by the MAL on preoperative CECT for esophagectomy.

## Background

Esophagectomy is one of the most invasive gastrointestinal surgical procedures and is associated with a range of postoperative morbidities [1]. Of these, anastomotic leakage remains an important complication with a reported incidence between 10 and 21.2% in large-scale studies [2, 3]. Long-term fasting is required once anastomotic leakage is identified, leading to prolonged hospital stays. In addition, anastomotic strictures may develop [4, 5] and impact on patient quality of life for many years postoperatively. Moreover, anastomotic leakage is a prognostic factor in patients undergoing esophagectomy for esophageal cancer [6, 7]. Therefore, the prevention of anastomotic leakage is a clinical priority for esophageal surgeons. Many factors, including gastric tube blood flow, nutrition, anastomosis method, and anastomotic location reportedly influence anastomotic outcomes [8, 9]. Recently, celiac artery stenosis due to arteriosclerosis has been reported to be associated with anastomotic leakage following Ivor–Lewis esophagectomy [10, 11]. Lainas et al. reported celiac artery stenosis due to compression by the median arcuate ligament (MAL), in addition to atherosclerotic celiac artery stenosis, was associated with necrosis of the gastric tube following Ivor–Lewis esophagectomy [12]. However, the management of the celiac artery stenosis due to MAL in patients with esophageal cancer undergoing gastric tube reconstruction has not been reported. Herein, we present the first reported case of esophageal cancer with celiac artery stenosis due to compression by the MAL successfully treated by McKeown esophagectomy and gastric tube reconstruction following prophylactic MAL release.

## Case presentation

**Fig. 1:**
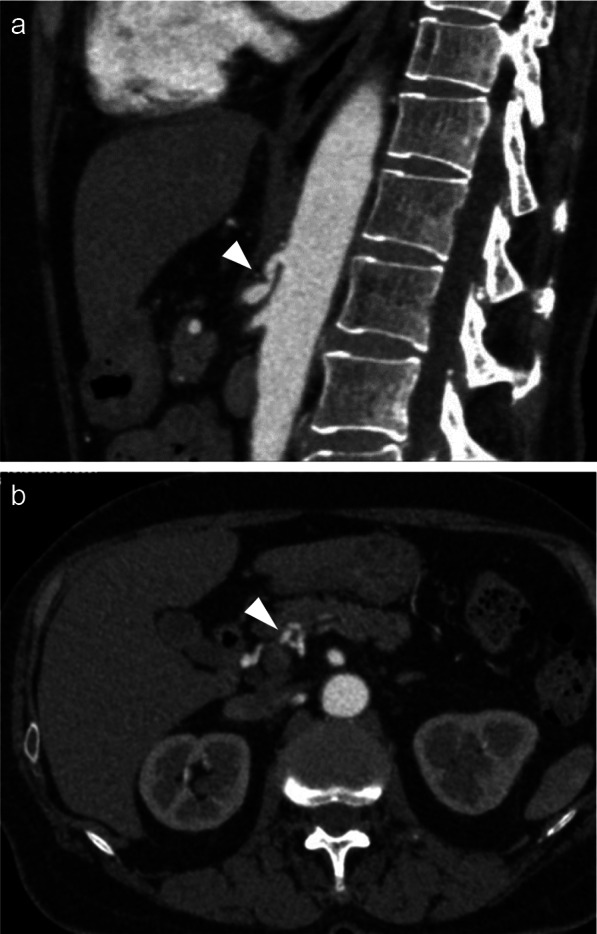
Preoperative abdominal contrast-enhanced computed tomography. **a** Sagittal image demonstrating celiac artery stenosis due to compression from the cranio-ventral side (arrowhead). **b** Axial image demonstrating collateral blood vessels around the pancreatic head (arrowhead)

A 72-year-old female patient with a history of radical hysterectomy and postoperative irradiation for cervical cancer 25 years prior was referred to our department for esophagectomy. The patient had received two courses of neoadjuvant chemotherapy with 5-FU and cisplatin for T2N0M0 squamous cell carcinoma of the middle esophagus. Preoperative contrast-enhanced computed tomography (CECT) showed celiac artery stenosis due to cranio-ventral compression, a finding typical of MAL syndrome (Fig. 1a). No aortic or celiac artery calcification was observed. The development of collateral arteries around the pancreatic head (i.e., arcade vessel from the superior mesenteric artery through the inferior pancreaticoduodenal artery to the gastroduodenal artery) was observed (Fig. 1b) without evidence of aneurysm formation. The patient reported no abdominal symptoms.

**Fig. 2:**
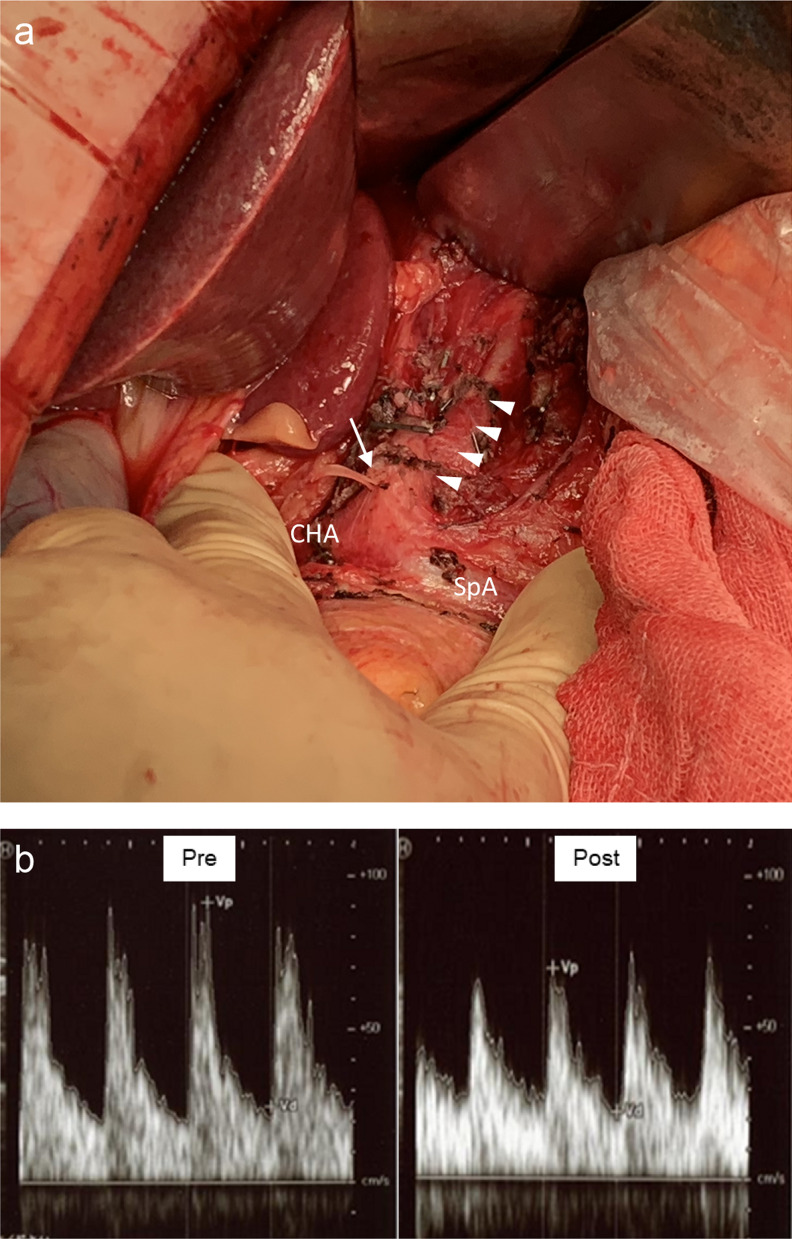
Intraoperative findings. **a** Median arcuate ligament (MAL) release was performed under laparotomy. Arrowheads indicate the celiac artery. The arrow indicates the stump of the left gastric artery. *CHA* common hepatic artery, *SPA* splenic artery. **b** Evaluation of celiac artery blood flow using Doppler sonography pre (left) and post (right) MAL release. Vp indicates systolic flow velocity. Vd indicates diastolic flow velocity

**Fig. 3:**
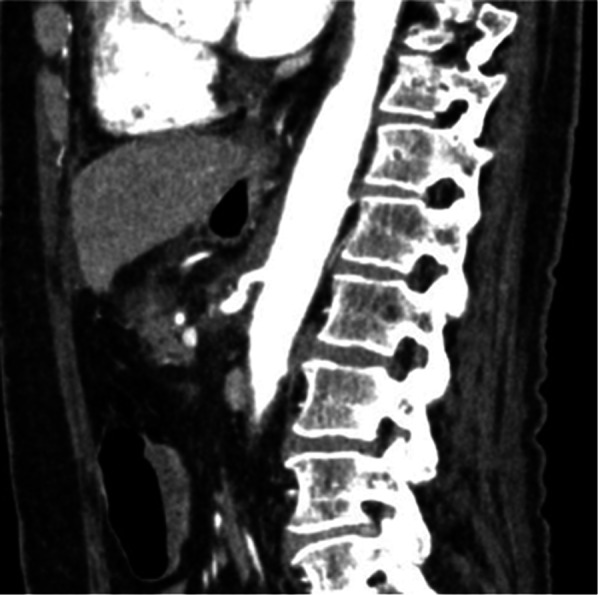
Abdominal contrast-enhanced computed tomography on postoperative day 7. Sagittal image demonstrating improved celiac artery patency

Robot-assisted thoracoscopic esophagectomy [13] with mediastinal lymphadenectomy [14] was performed in the prone position, followed by gastric mobilization and supra-pancreatic lymphadenectomy by open laparotomy in the supine position. Then, a gastric tube was created using linear staplers and the lesion was excised en bloc. Subsequently, the MAL was cut and released to expose the celiac artery (Fig. 2a). Celiac artery blood flow was assessed by the pulsatility index (PI), which provides information pertaining to down-stream vascular resistance, calculated from the difference between systolic and diastolic flow velocities divided by the mean velocity using intraoperative Doppler sonography [15, 16]. The PI decreased from 1.54 to 1.18 after the MAL release, indicating decreased vascular resistance and improved celiac artery patency (Fig. 2b). Perfusion of the gastric tube was evaluated in a non-quantitative way using an indocyanine green (ICG) fluorescence imaging system as our routine practice. In the present case, it was as good as usual even before the MAL release and there was no apparent difference observed between before and after the MAL release. The cervical esophagogastric anastomosis was performed using the triangulating stapling technique [17] following cervical lymphadenectomy. CECT imaging on postoperative day (POD) 7 showed improved celiac artery patency (Fig. 3). The postoperative course was uneventful except for Clavien–Dindo [18] Grade I left recurrent nerve palsy and the patient was discharged on POD 21. No recurrence was observed up to 2 years postoperatively.

## Discussion

The MAL is a band of fibrous tissue that connects the bilateral diaphragmatic crura surrounding the aortic hiatus. Individuals with a high origin of the celiac artery or lower insertion of the diaphragm are more prone to compression of the celiac artery [19]. It is reported that in 10% to 24% of the population, the MAL crosses the aorta at a lower level, resulting in compression of the celiac artery [20]. Although the incidence of MAL syndrome in the population is not well understood, it is more prevalent in women (4:1 ratio) between the ages of 30 to 50 years and in patients with a thin body habitus [21]. In the present case, we successfully performed subtotal esophagectomy with prophylactic MAL release for esophageal cancer with celiac artery stenosis. This is the first report of prophylactic MAL release for celiac artery stenosis in a patient undergoing gastric tube reconstruction following esophagectomy.

The gastric tube blood flow is predominantly from the right gastroepiploic artery through the gastroduodenal artery, with little contribution from the right gastric artery [22]. The gastroduodenal artery normally receives its blood flow from the celiac artery through the common hepatic artery; however, in the presence of celiac artery stenosis, it receives its blood flow from the superior mesenteric artery [23, 24]. Therefore, in cases of gastric tube reconstruction following esophagectomy for patients with celiac artery stenosis, correction of celiac artery stenosis is controversial. Regarding this issue, Lammerts et al. reported the use of preoperative percutaneous stent angioplasty followed by Ivor–Lewis esophagectomy for esophageal cancer with atherosclerotic celiac artery stenosis [25]. Therapeutic intervention for celiac artery stenosis is frequently reported in cases of pancreaticoduodenectomy [26, 27]. Sugae et al. classified celiac artery stenosis due to MAL into three types according to the degree and length of stenosis based on CT angiography findings in patients undergoing pancreaticoduodenectomy [27]. They concluded MAL release was required in cases of moderate stenosis (50–80% stenosis and 3–8 mm stenosis length), and collateral preservation or arterial reconstruction was required in cases of severe stenosis (> 80% stenosis and > 8 mm stenosis length). In the present case, CECT showed 75% stenosis with a stenosis length of 4.5 mm, which corresponded to moderate stenosis. Accordingly, MAL release was, expected to improve celiac artery blood flow. Recently, celiac artery stenosis due to MAL or arteriosclerosis has been reported to be associated with necrosis of the gastric tube or anastomotic leakage following Ivor–Lewis esophagectomy [10–12]. Among these reports, Chang et al. reported that the mean degree of celiac artery stenosis in patients with anastomotic leakage was 44.4% [10], and Brinkmann et al. reported that the median degree of celiac artery stenosis in patients with anastomotic leakage was 50.0% [11]. Moreover, McKeown esophagectomy with three fields’ lymph node dissection, which is standard procedure in Japan [28], requires a higher anastomosis than Ivor–Lewis esophagectomy. Taken together, the risk of postoperative anastomotic complication would be higher in the present case. In addition, as the present case had already developed collateral arteries around the pancreatic head, we performed prophylactic MAL release to ensure favorable short-term anastomotic outcomes and prevent future aneurysmal rupture leading to gastric tube necrosis.

As for the operative strategy for a case of esophageal cancer with celiac artery stenosis, if the initial ICG fluorescence imaging of the gastric tube is satisfactory, the MAL release would be the prophylactic procedure anyway. On the contrary, if there are any discrepancies between the decrease of the PI and the change of the ICG fluorescence images even after thorough dissection of the MAL in a patient with poor initial ICG imaging, the sufficient perfusion of the gastric tube would not be guaranteed. In such case, we consider either revascularization of the gastric tube at the neck or two-stage reconstruction.

In elective cases with MAL syndrome, we typically use the laparoscopic approach in a hybrid operating room to confirm hemodynamic alterations using concomitant angiography [29], and our team has sufficient experience of laparoscopic gastrectomy even in patients with a history of previous laparotomy [30]. Therefore, we considered laparoscopic gastric mobilization and MAL release to be feasible in the present case. However, considering the operative duration and limited choice of operating rooms for robotic surgery, we opted to use an open approach and evaluate celiac artery flow using Doppler sonography, which represents a method of real-time intraoperative monitoring [31].

## Conclusions

In patients with celiac artery stenosis due to compression by the MAL on preoperative CECT for esophagectomy, preservation of gastric tube blood flow should be ensured prior to reconstruction. Prophylactic MAL release may be considered in cases, where celiac artery stenosis may increase risk of anastomotic complications.

## Data Availability

Not applicable.

## Abbreviations

MAL: Median arcuate ligament
CECT: Computed tomography
PI: Pulsatility index
POD: Postoperative day
